# Supplementary material for: Histone H3.3 lysine 9 and 27 control repressive chromatin at cryptic enhancers and bivalent promoters
Source: Nat Commun. 2024 Aug 30;15:7557. doi: 10.1038/s41467-024-51785-w (PMC11364623; doi:10.1038/s41467-024-51785-w)
Supplement: Supplementary file 1 — Supplementary Information [file 41467_2024_51785_MOESM1_ESM.pdf]

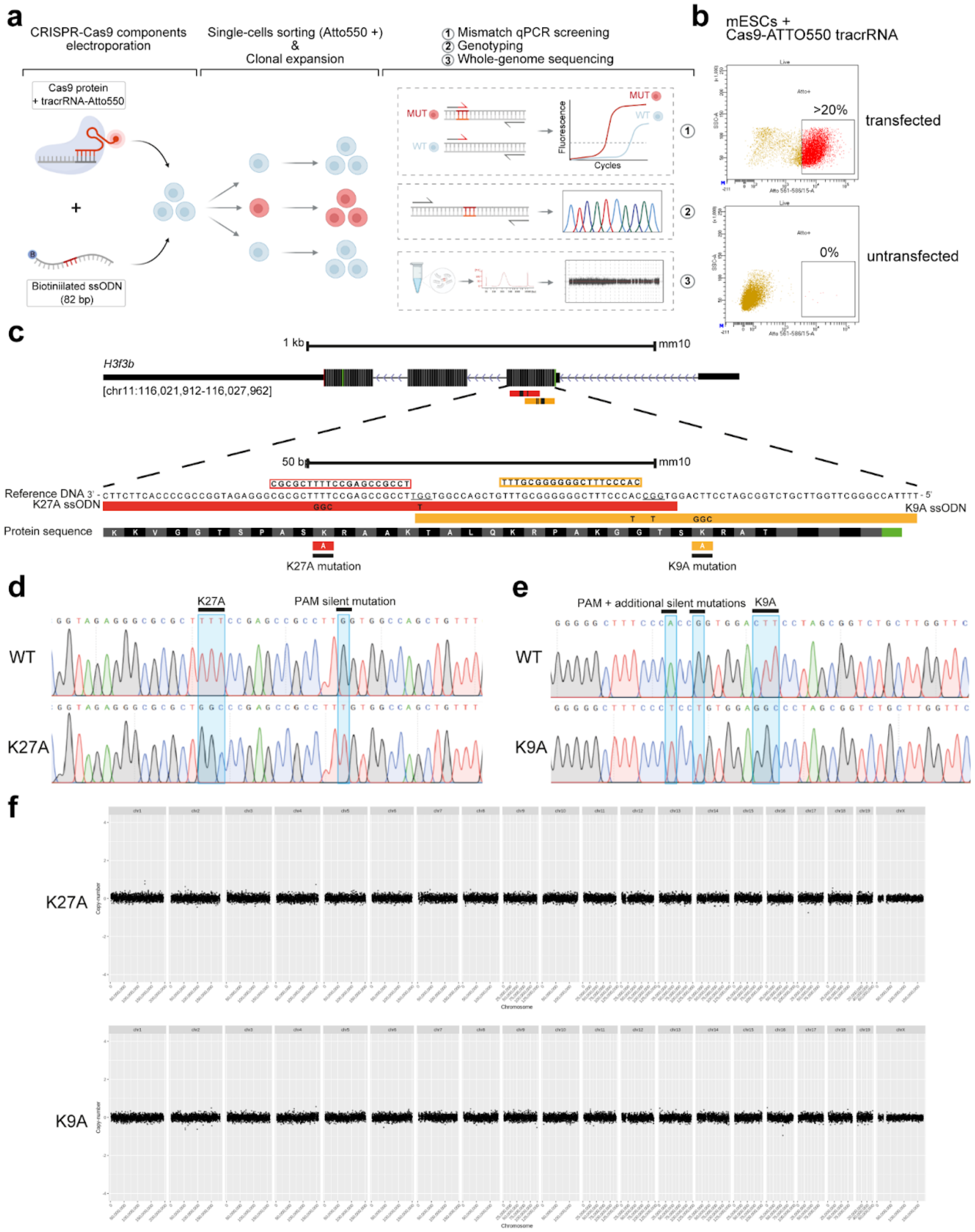

Supplementary Figure 1: CRISPR-Cas9 editing strategy and chromosomal integrity assessment for the edited clones. (a) Schematic of the Cas9 RNP editing workflow. **(b)** Flow cytometry analysis displays mESCs transfection efficiency with Cas9-tracrRNA-ATTO550 (about 20% or higher - top). Untransfected cells subjected to Cas9 RNP complex but not transfected were used to set the gates for flow cytometry analysis (bottom). **(c)** Schematic with guide RNAs and ssODN designed to introduce K27A (red) and K9A (orange) mutations in the *H3f3b* locus. **(d-e)** Representative Sanger sequencing chromatograms showing the comparison between wild-type and K27A (d) or K9A (e) mESCs at the *H3f3b* endogenous locus. **(f)** Representative whole-genome plots showing the copy-number difference of 100 kb genomic bins between edited clones and the parental control line. Panel 1a created with BioRender.com released under a Creative Commons Attribution-NonCommercial-NoDerivs 4.0 International license.

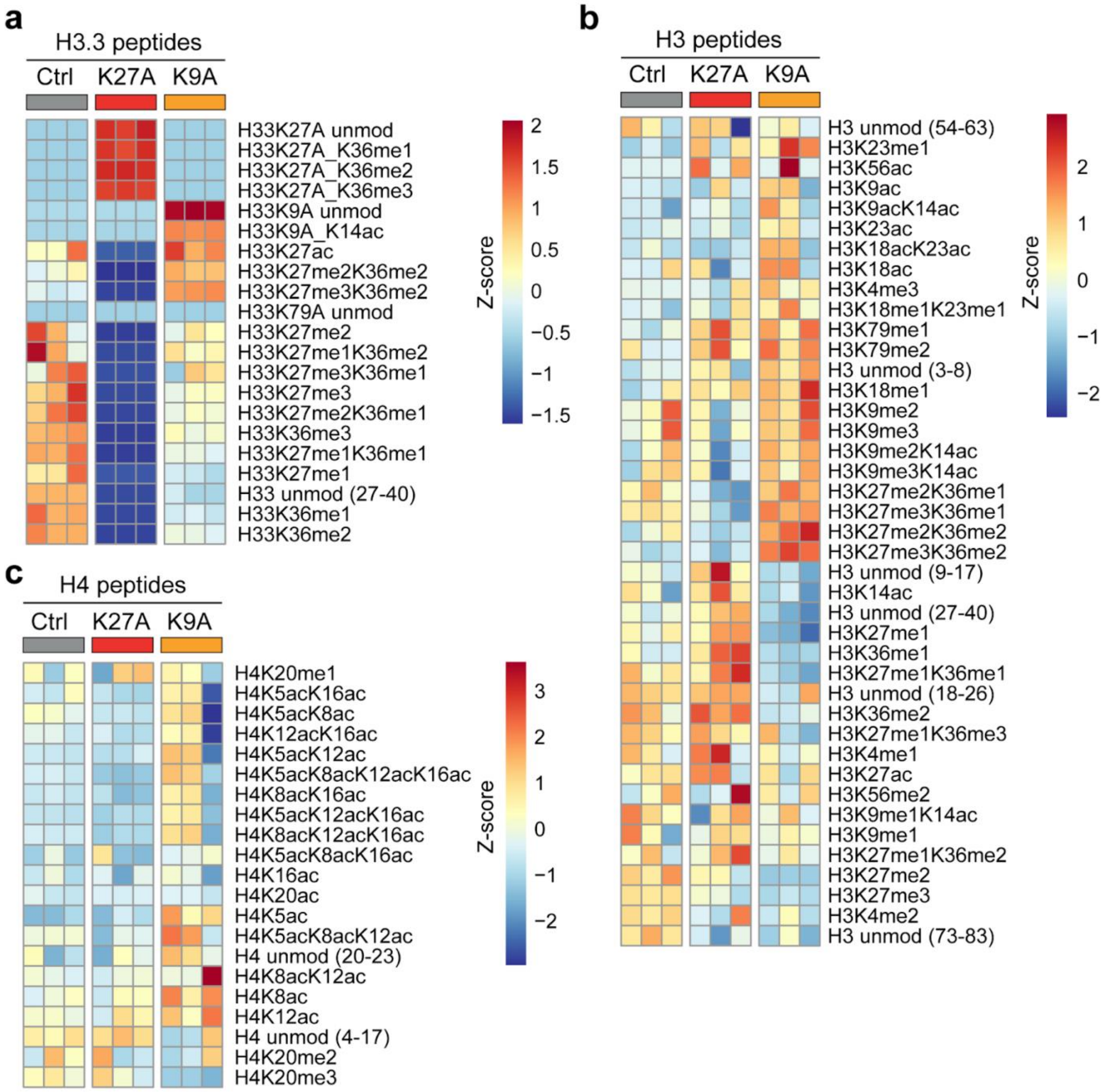

Supplementary Figure 2: Histone modifications middle-down mass spectrometry analysis. Heatmaps displaying the relative abundance (row z-score) of individual or combinatorial post-translational modifications of histone H3.3 **(a)**, H3 **(b)** or H4 **(c)**. Lysine-to-alanine mutated H3.3 peptides were used as controls in panel "a". The peptides length was omitted for clarity from the figure and is reported here: H3K4 (3-8); H3K9/K14 (9-17); H3K18/K23 (18-26); H3K27/K36 (27-40); H3K56 (54-63); H3K79 (73-83); H4K20 (20-23); H4K5/K8/K12/K16 (4-17); H4K20 (20-23). Source data are provided as a Source Data file.

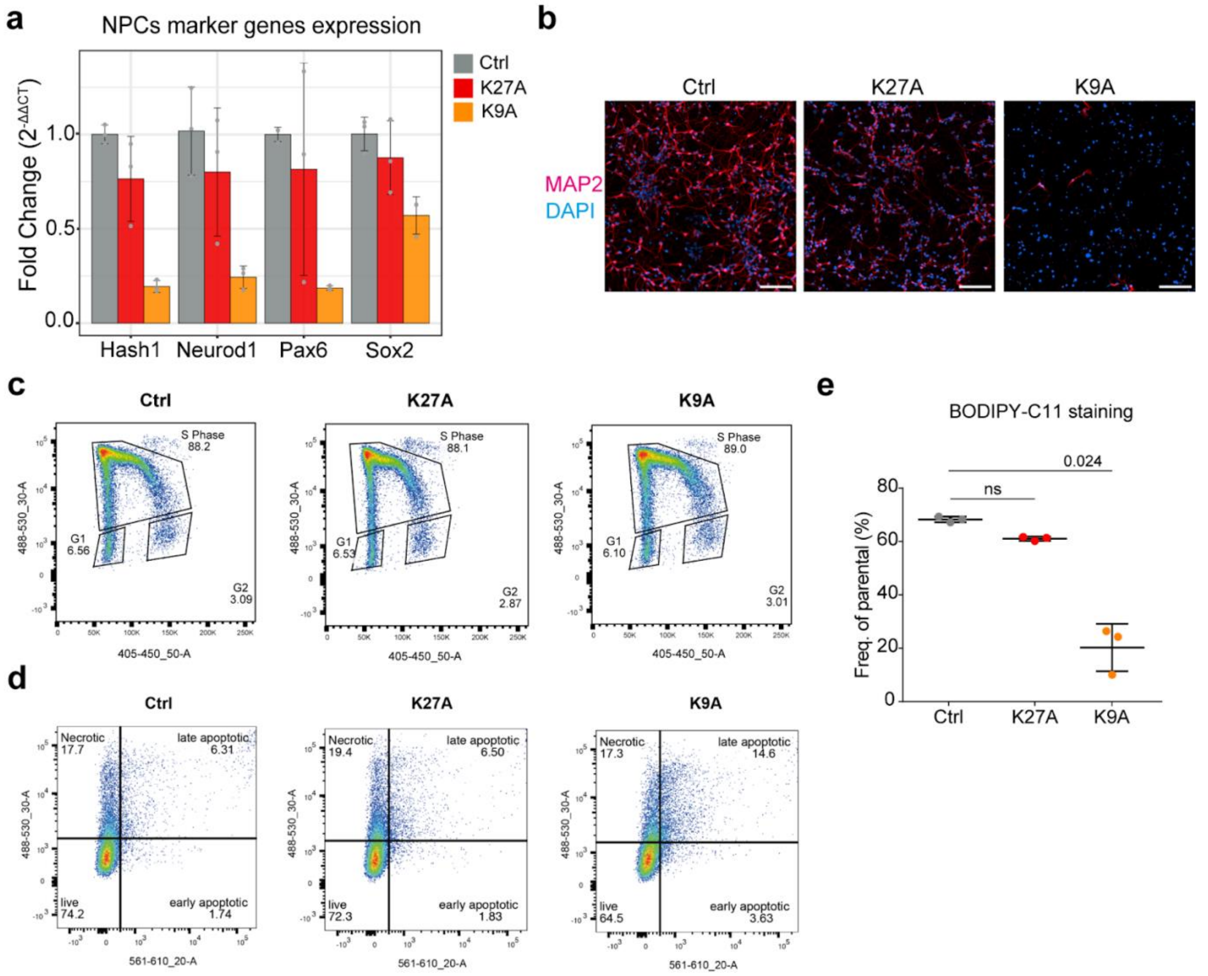

Supplementary Figure 3: Phenotypic characterization of H3.3 K27A and K9A mESCs. (a) RT-qPCR results for neuronal progenitor cell markers (day 8) in control, K9A, and K27A (n=3 independent clonal lines/condition). Data are the mean ± standard deviation of gene expression values ($2^{-\Delta\Delta Ct}$) normalized to Rpl13 housekeeping gene and control. **(b)** Merged immunofluorescence images of neurons on day 12 of the *in-vitro* differentiation, stained with antibodies against MAP2 and with DAPI to detect nuclei. Scale bar 100 µm. **(c)** Representative plots of flow cytometry analysis displaying gating strategy for measuring 5-ethynyl-2'-deoxyuridine (EdU) incorporation in control, K27A, and K9A mESCs. **(d)** Representative plots of flow cytometry analysis upon Annexin V staining for control, K27A, and K9A mESCs. **(e)** Levels of lipid peroxides measured in control, K27A, and K9A mESCs using BODIPY-C11 staining and flow-cytometry analysis. *P* value: two-sided unpaired t-test. Source data are provided as a Source Data file.

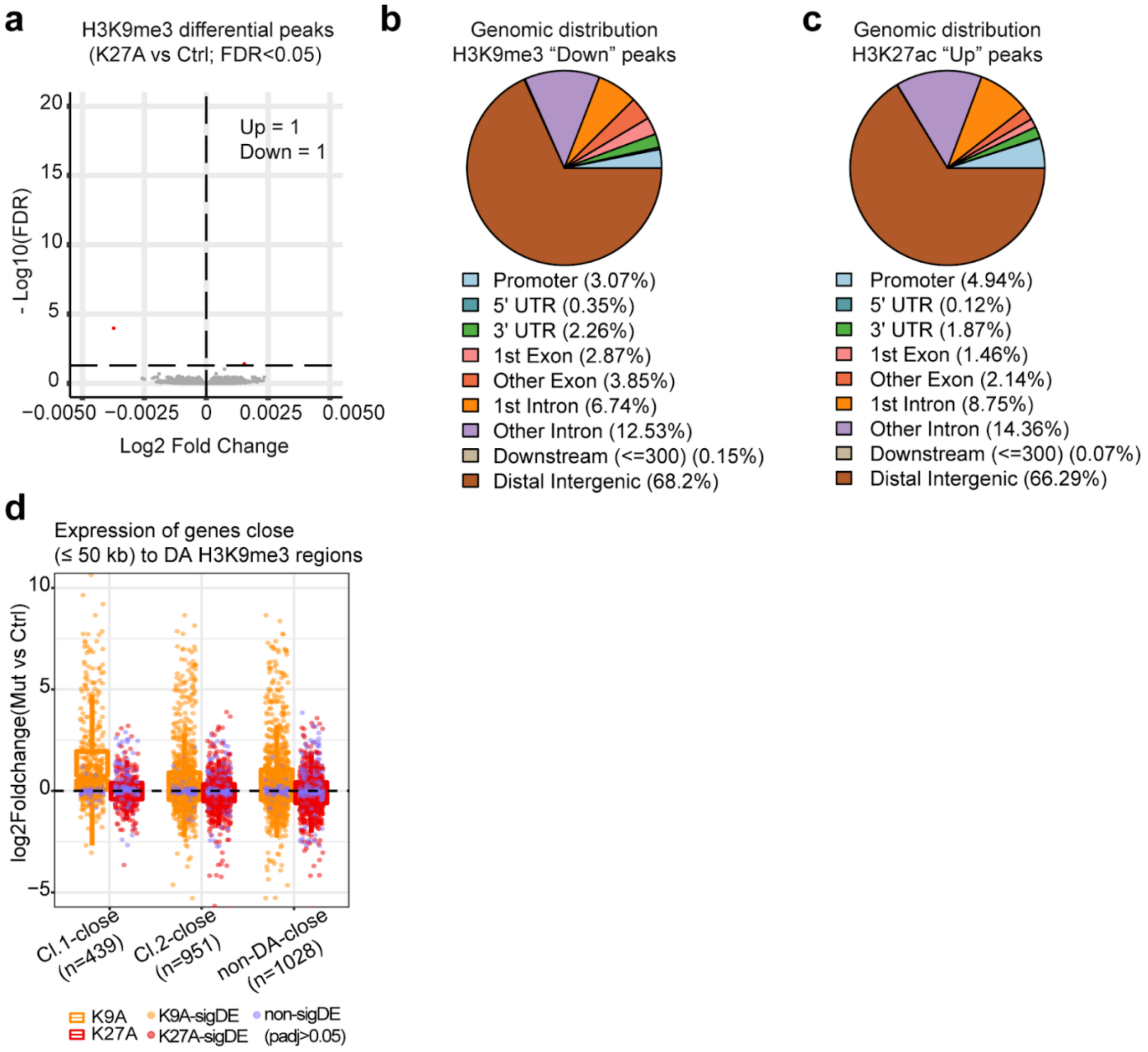

Supplementary Figure 4: Analysis of DA-H3K9me3 regions. (a) Volcano plots showing differential H3K9me3 ChIP-seq analysis in K27A. **(b)** Genomic annotation of the H3K9me3 regions with significantly reduced signal in K9A mESCs. Promoter defined as TSS ± 1 kb. **(c)** Genomic annotation of the H3K27ac regions with significantly increased signal in K9A mESCs. Promoter defined as TSS ± 1 kb. **(d)** Expression of genes located within 50 kb from DA-H3K9me3 regions; individual genes (data points) are colored to indicate if the differential expression is significant (orange/red) or not (purple). Source data are provided as a Source Data file.

a

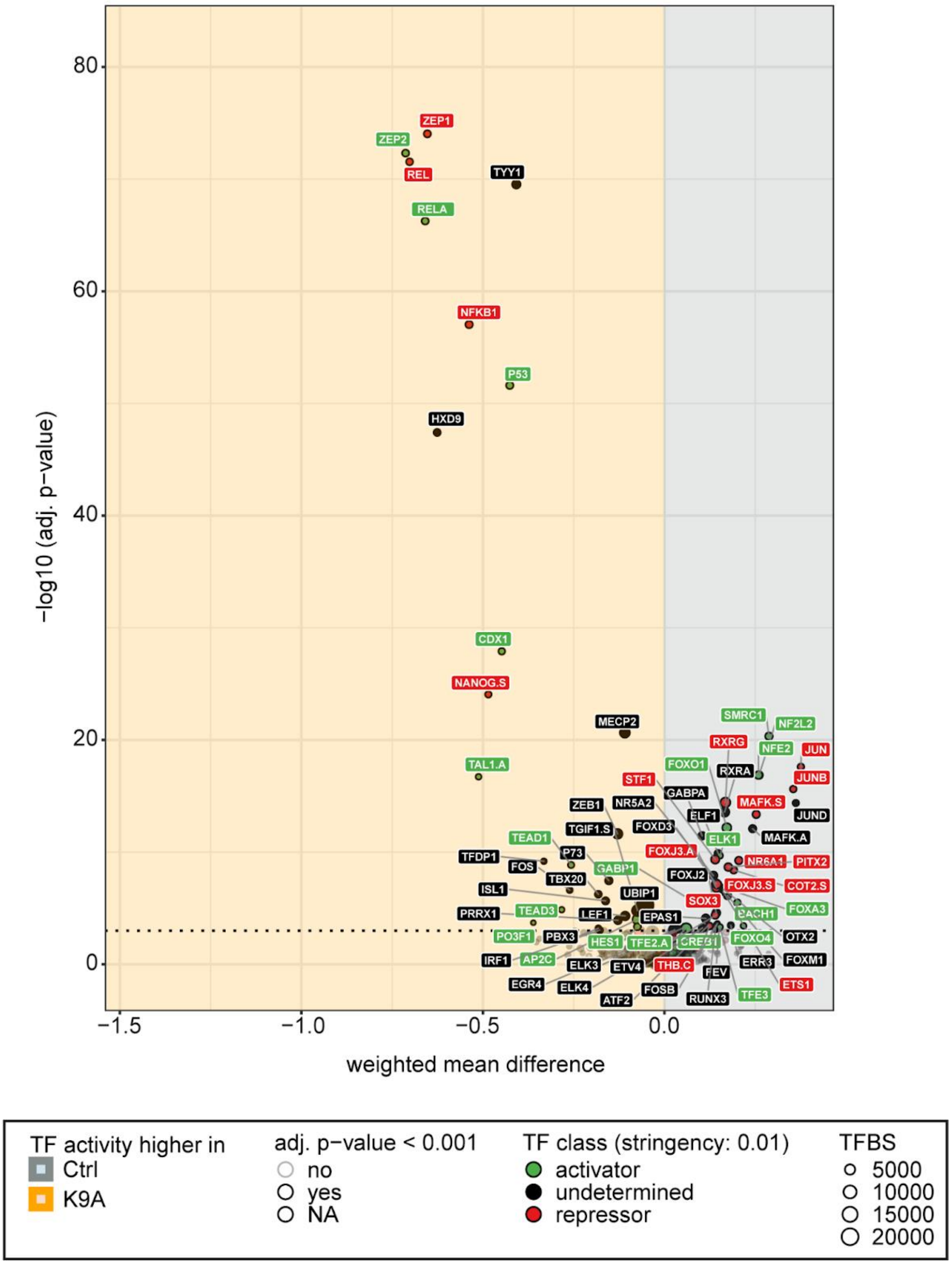

Supplementary Figure 5: diffTF analysis for K9A mESCs. (a) Volcano plot showing TFs with higher activity in K9A (left – orange) or in control (right – grey). TFs are classified as activators (green) or repressors (red). Only expressed TFs are displayed.

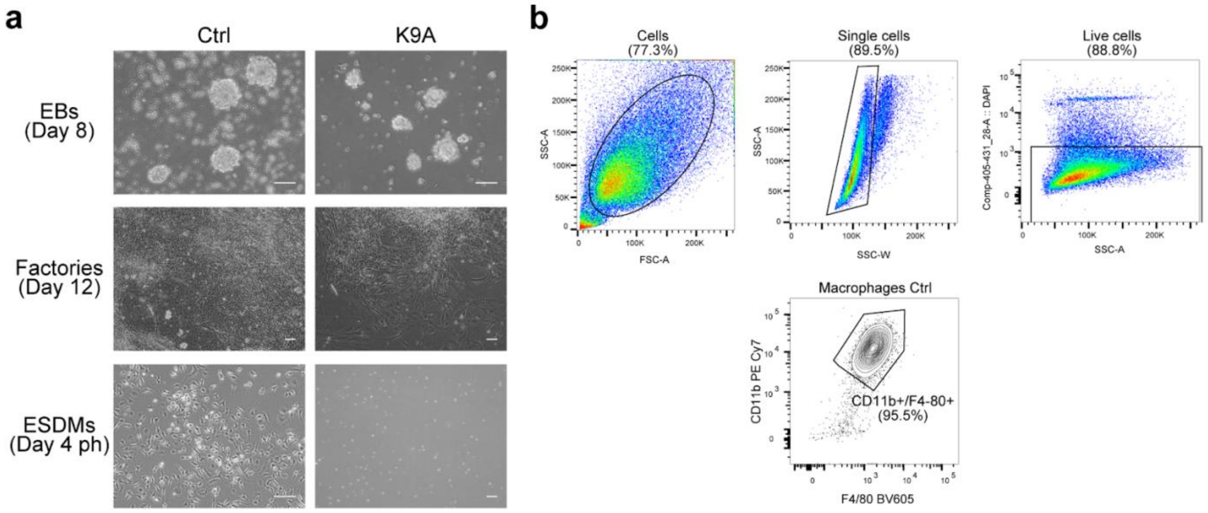

Supplementary Figure 6: *In-vitro* macrophage differentiation. (a) Representative bright-field images of embryoid bodies (EBs, day 8), Factories (day 12), and ES cells-derived macrophages (ESDMs, day 4 post-harvesting) were obtained from control and K9A mESCs. **(b)** Representative plots of flow cytometry analysis displaying gating strategy for measuring the percentage of control mESCs-derived macrophages, positively stained with anti-CD11b and anti-F4/80 antibodies.

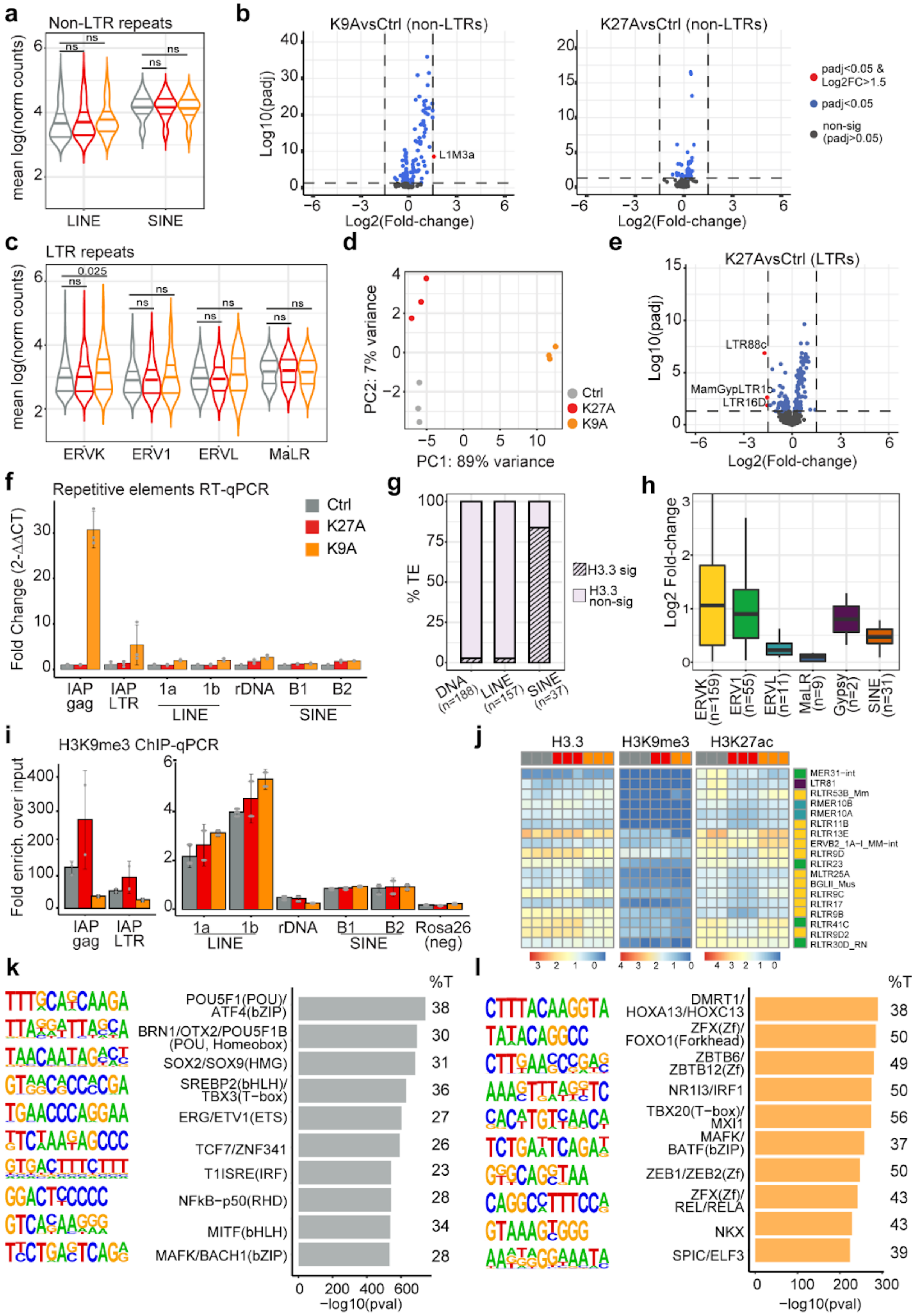

Supplementary Figure 7: Transposable elements analysis. (a) Violinplots displaying expression of highly expressed non-LTR repeats (i.e., average log-transformed normalized count values above median), in control and mutant mESCs. Total repeats accounted for n=94, of which n=60 LINE and n=34 SINE. P-value: two-sided unpaired t-test (ns: p-value>0.05). **(b)** Volcano plots showing differential non-LTR repeats

expression in K9A (left) and K27A (right) mESCs (padj<0.05 & abs(log2FC)>1.5). **(c)** Violinplots displaying expression of highly expressed ERV families (i.e., average log-transformed normalized count values above median) in control and mutant mESCs. Total ERVs accounted for n=297, of which n=158 ERVK (53%;), n=42 ERV1 (14%), n=28 ERVL (9.4%), and n=69 MaLR (23%). P-value: two-sided unpaired t-test (ns: p-value>0.05). **(d)** Principal component analysis of LTRs expression. **(e)** Volcano plot showing differential ERVs expression in K27A mESCs (padj<0.05 & abs(log2FC)>1.5). **(f)** RT-qPCR using primers for selected repeat elements. **(g)** Stacked barplot indicating the percentage of transposable elements with or without significant (pval<0.01) H3.3 enrichment over input in control mESCs. **(h)** Boxplot of H3.3 enrichment (log2FC over input) at ERV families and SINE elements, displaying significant H3.3 signal (from panels S7g and Fig. 5d). **(i)** H3K9me3 ChIP-qPCR with primers for ERVs (left) or other TEs (right). Rosa26 locus was used as a negative control for H3K9me3 enrichment. **(j)** Examples of ERV subfamilies displaying non-significant H3K9me3 enrichment and concomitant significant H3K27ac enrichment in control mESCs without notable further activation in K9A mESCs. **(k)** TF motifs enriched at ERVs marked by high H3K27ac (i.e., enrichment over input above median) in control and K9A mESCs. **(l)** TF motifs at ERVs marked by high H3K27ac (i.e., enrichment over input above median) exclusively in K9A mESCs. Analysis performed with Homer de-novo enrichment ("–size given"). %T: percentage of targets with motif. Source data are provided as a Source Data file.

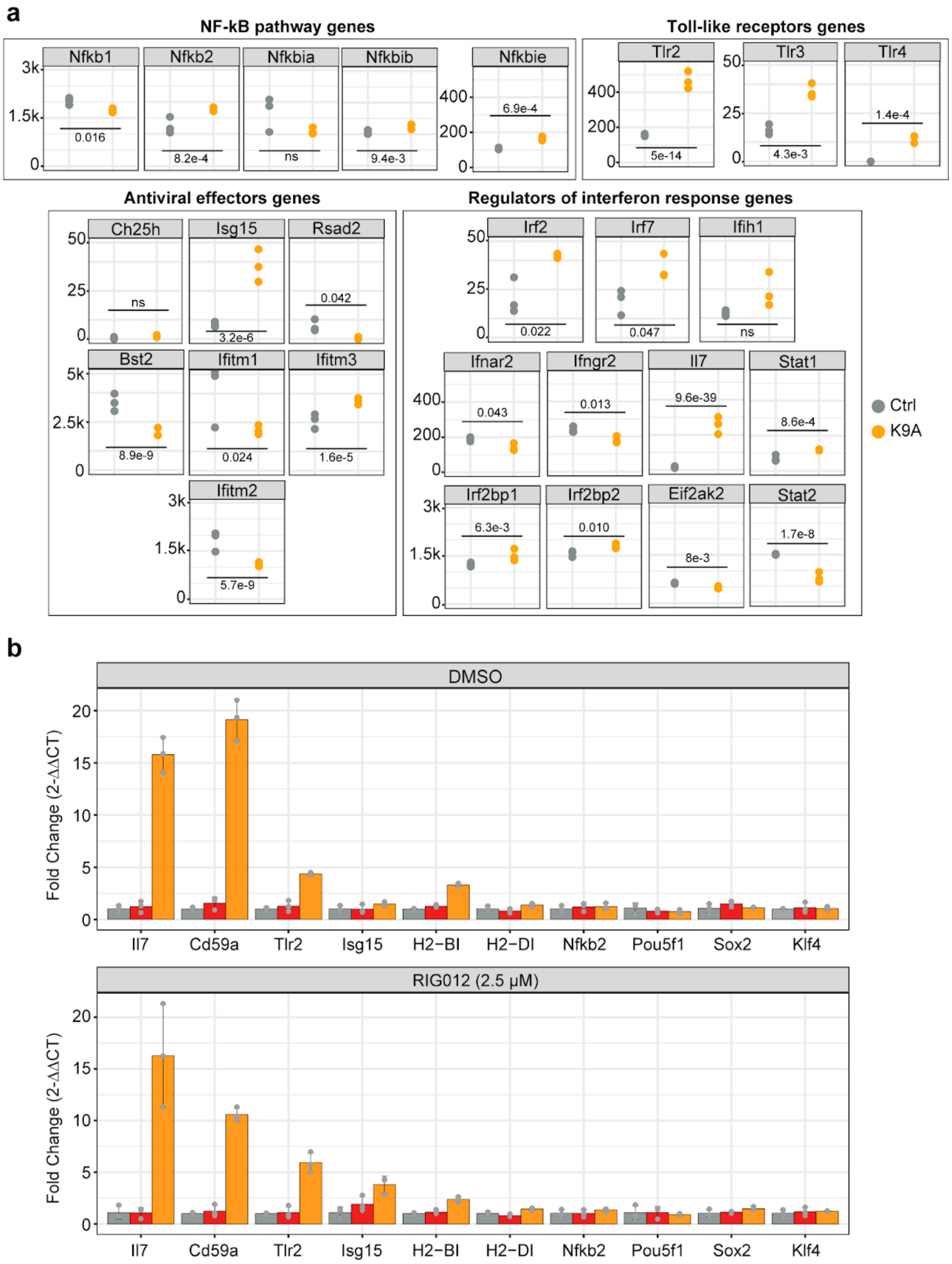

Supplementary Figure 8: Evaluation of viral mimicry involvement in immune-related gene expression. (a) Normalized mRNA-seq counts for selected interferon-stimulated genes in control and K9A mESCs. Genes are divided in four groups: NF-kB pathway genes (Nfkb1/p50; Nfkb2/p65; Nfkbia/IkB-alpha; Nfkbib/IkB-beta; Nfkbie/IkB-epsilon), Toll-like receptors genes, Antiviral effectors genes (Ch25h; Isg15; Rsad2/Viperin; Bst2/Tetherin; Ifitm1-2-3) and Regulators of interferon response genes (Irf2-7; Ifih1/Mda5; Ifnar2; Ifngr2; Il7; Irf2bp1-2; Eif2ak2/Pkr; Stat1-2). Significant differences were calculated with DESeq2, and p-values adjusted with Benjamini Hochberg's correction (ns: p-adj>0.05). **(b)** Real-time qPCR for immune genes and pluripotency factor genes upon treatment of DMSO vehicle or of 2.5 µM RIG012 inhibitor in control and mutant mESCs (n=3 independent clonal lines/condition). Data are the mean ± standard deviation of gene expression values (2−ΔΔCt) normalized to Rpl13 housekeeping gene and control in each treatment group. Source data are provided as a Source Data file.

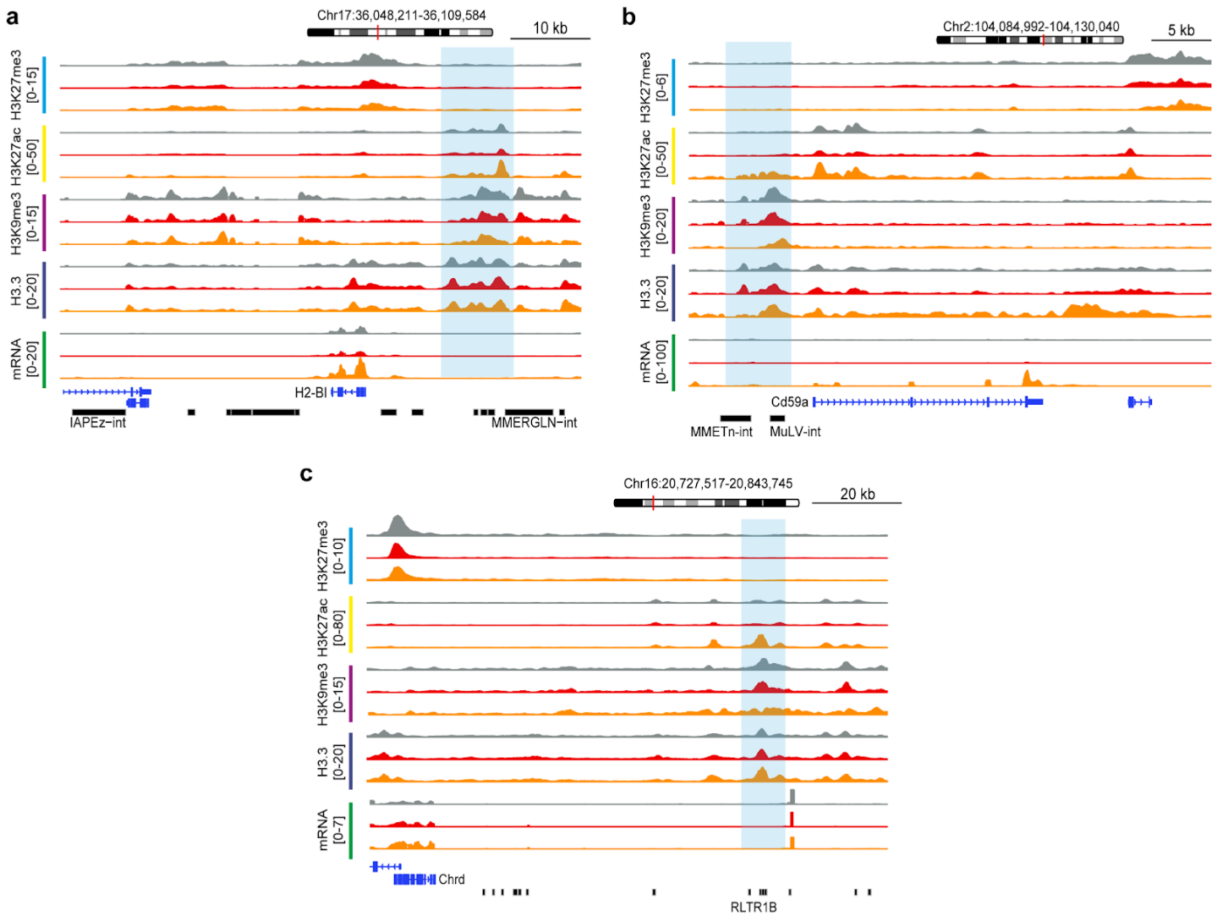

Supplementary Figure 9: Genome browser snapshots of representative DA-H3K9me3 regions. (a-d) Genome browser snapshot of a representative Cluster 1 and Cluster 2 DA-H3K9me3 region (highlighted), linked through the GRN to the H2-Bl **(a)**, Cd59a **(b)** and Chrd **(c)** genes. H3K27me3, H3K27ac, H3K9me3, H3.3, and mRNA-seq tracks are shown. For ERV annotation, only the name of the ERV located within the DA-H3K9me3 region is shown.

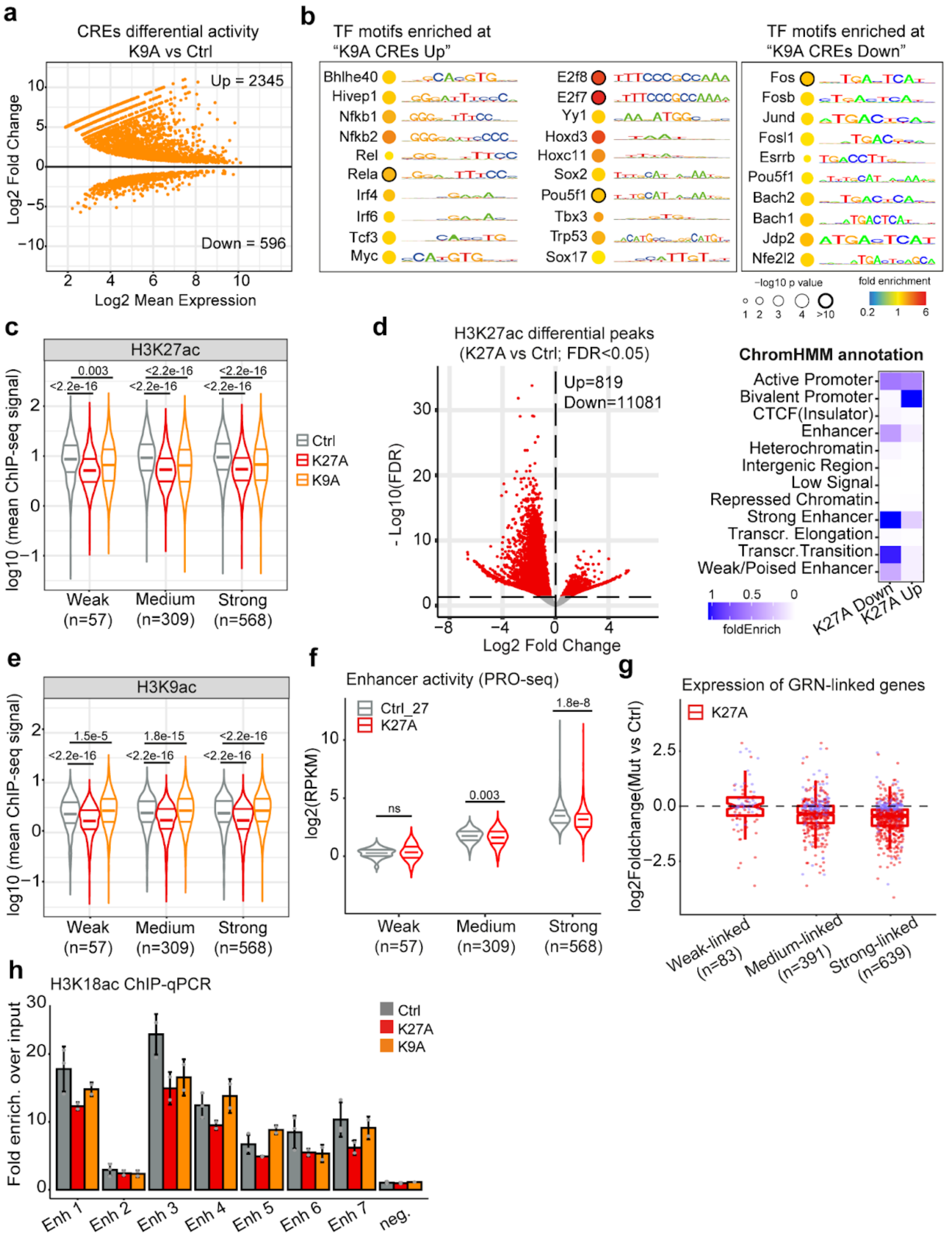

Supplementary Figure 10: Enhancers analysis. (a) MA-plot showing differentially active CREs, according to tfTarget analysis (retained CREs as significant with padj<0.01). **(b)** Selected TF motifs significantly enriched at CREs with increased (left) or decreased (right) activity in K9A mESCs. **(c)** Violin plots showing H3K27ac ChIP-seq signal at peak center ± 1 kb of enhancers retained in the GRN. P-value: two-sided unpaired t-test (ns: p-value>0.05). **(d)** Volcano plot showing differential H3K27ac ChIP-seq peaks in K27A vs. control mESCs and ChromHMM annotation of DA-H3K27ac regions. **(e)** Violin plots showing H3K9ac ChIP-seq signal at peak center ± 1 kb of enhancers retained in the GRN. P-value: two-sided unpaired t-test (ns: p-value>0.05). **(f)** PRO-seq signal at Weak, Medium, and Strong dREG enhancers included in the GRN for K27A and control mESCs. Log2(RPKM) values are reported. P-value: two-sided unpaired t-test (ns: p-value>0.05) **(g)** Expression of DEGs connected to dREG enhancers through the GRN; log2(fold-change) values calculated with DESeq2 are plotted, and individual genes (data points) are colored to indicate if the differential expression is significant (red) or not (purple). In panels "c", "d" and "f", significance was calculated with two-sided unpaired t-test (ns: p-value>0.05). **(h)** H3K18ac ChIP-qPCR at selected Strong enhancers regions. The relative enrichment over the input is reported, and the data are mean ± standard deviation (n=3 replicates Ctrl; n=2 replicates mutants). Source data are provided as a Source Data file.

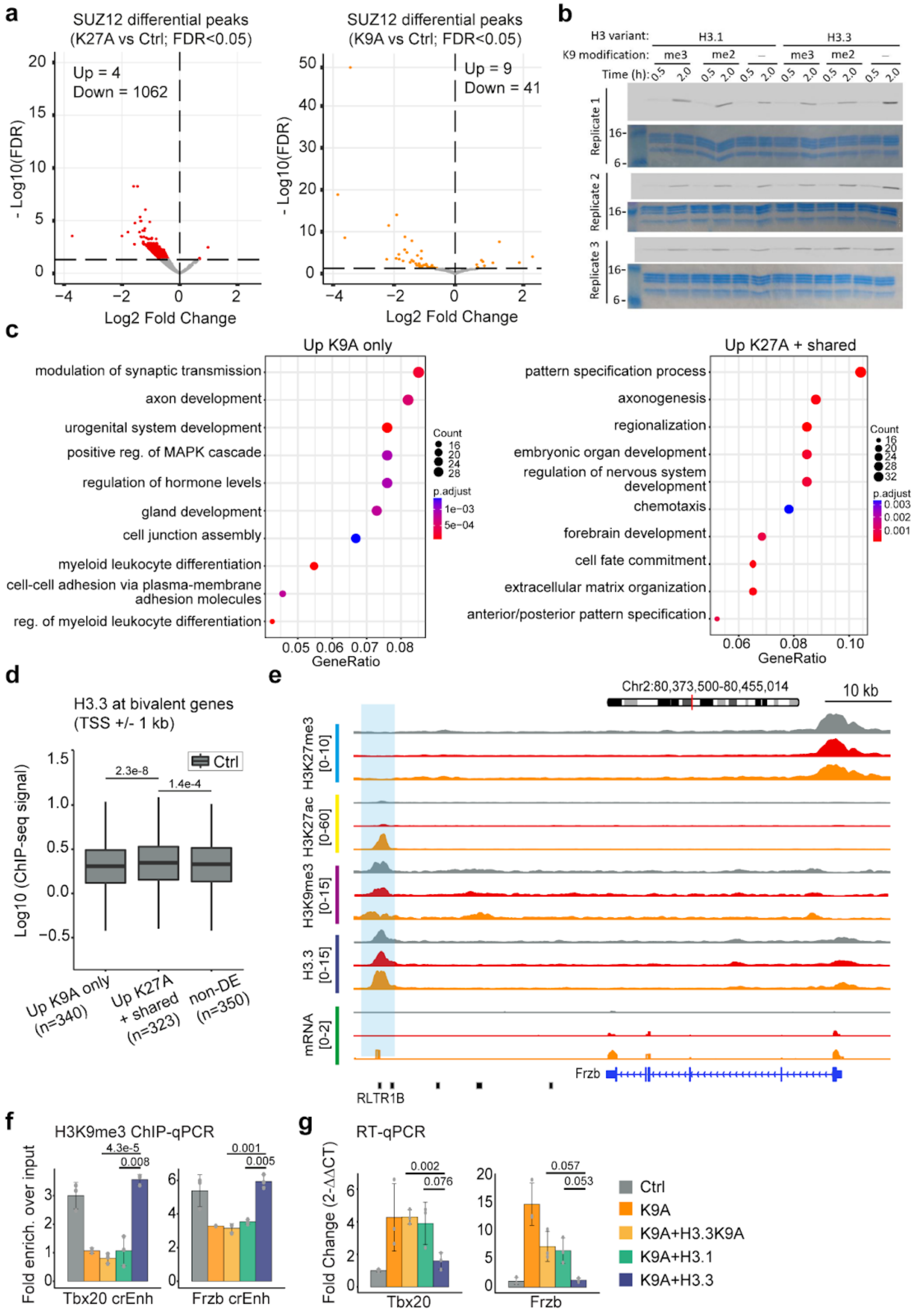

Supplementary Figure 11: Effect of H3.3 K27A and K9A mutations on PRC2. (a) Volcano plots showing differential SUZ12 ChIP-seq peaks in K27A (left) or K9A (right) versus control (consensus peakset with n=3356 peaks). **(b)** Radiograms were used to calculate the mean values reported in Fig. 7g for the PRC2 methyltransferase assay. **(c)** Gene ontology enrichment analysis of bivalent genes upregulated only in

K9A (left) or in K27A/K27A+K9A mESCs (right). P.adjust: p-values of significant GO terms adjusted for multiple comparisons. **(d)** H3.3 ChIP-seq signal in control mESCs, at the promoter regions of bivalent genes, upregulated only in K9A or K27A/K27A+K9A. A group of comparable size with non-significantly differentially expressed bivalent genes was randomly selected for comparison. P-value: two-sided unpaired t-test. **(e)** Genome browser snapshot of a representative Cluster 2 DA-H3K9me3 region (highlighted), linked through the GRN to the Frzb gene. For ERVs annotation, only the name of the ERV located within the DA-H3K9me3 region is shown; from left to right, the other elements are RMER10A, RLTR20B2, RLTR13B1, and RMER15-int. **(f)** H3K9me3 ChIP-qPCR at selected cryptic enhancer regions, connected through the GRN to Tbx20 and Frzb genes, in control, K9A and K9A mESCs stably expressing H3.3K9A, H3.1 or H3.3WT. The relative enrichment over the input is reported, and the data are mean ± standard deviation (n=3 independent replicates). crEnh: cryptic enhancer. **(g)** RT-qPCR results for Tbx20 and Frzb genes in control, K9A, and K9A cells stably expressing H3.3K9A, H3.1, or H3.3WT. P-value: two-sided unpaired t-test. Source data are provided as a Source Data file.
