# Supplementary material for: Histone H3.3 lysine 9 and 27 control repressive chromatin at cryptic enhancers and bivalent promoters
Source: Nat Commun. 2024 Aug 30;15:7557. doi: 10.1038/s41467-024-51785-w (PMC11364623; doi:10.1038/s41467-024-51785-w)
Supplement: Supplementary file 3 — Description of Additional Supplementary Files [file 41467_2024_51785_MOESM3_ESM.pdf]

Description of Additional Supplementary Files

File Name: Supplementary Data 1
Description: The file contains sequences of single-guide RNAs and single-strand oligonucleotides used in CRISPR-Cas9 editing experiments to introduce K27A and K9A mutations in the endogenous *H3f3b* locus.

File Name: Supplementary Data 2
Description: The file contains sequences of forward and reverse primers used for real-time quantitative PCR (sheet #1) and ChIP-qPCR (sheet #2) experiments.
