# Supplementary material for: Histone H3.3 lysine 9 and 27 control repressive chromatin at cryptic enhancers and bivalent promoters
Source: Nat Commun. 2024 Aug 30;15:7557. doi: 10.1038/s41467-024-51785-w (PMC11364623; doi:10.1038/s41467-024-51785-w)
Supplement: Supplementary file 6 — Reporting Summary [file 41467_2024_51785_MOESM6_ESM.pdf]

nature portfolio

Corresponding author(s): Kyung-Min Noh

Last updated by author(s): Jul 15, 2024

Reporting Summary

Nature Portfolio wishes to improve the reproducibility of the work that we publish. This form provides structure for consistency and transparency in reporting. For further information on Nature Portfolio policies, see our Editorial Policies and the Editorial Policy Checklist.

Statistics

For all statistical analyses, confirm that the following items are present in the figure legend, table legend, main text, or Methods section.

n/a	Confirmed	
☐	☒	The exact sample size (*n*) for each experimental group/condition, given as a discrete number and unit of measurement
☐	☒	A statement on whether measurements were taken from distinct samples or whether the same sample was measured repeatedly
☐	☒	The statistical test(s) used AND whether they are one- or two-sided<br>*Only common tests should be described solely by name; describe more complex techniques in the Methods section.*
☐	☒	A description of all covariates tested
☐	☒	A description of any assumptions or corrections, such as tests of normality and adjustment for multiple comparisons
☐	☒	A full description of the statistical parameters including central tendency (e.g. means) or other basic estimates (e.g. regression coefficient) AND variation (e.g. standard deviation) or associated estimates of uncertainty (e.g. confidence intervals)
☐	☒	For null hypothesis testing, the test statistic (e.g. *F*, *t*, *r*) with confidence intervals, effect sizes, degrees of freedom and *P* value noted<br>*Give P values as exact values whenever suitable.*
☒	☐	For Bayesian analysis, information on the choice of priors and Markov chain Monte Carlo settings
☐	☒	For hierarchical and complex designs, identification of the appropriate level for tests and full reporting of outcomes
☐	☒	Estimates of effect sizes (e.g. Cohen's *d*, Pearson's *r*), indicating how they were calculated

Our web collection on statistics for biologists contains articles on many of the points above.

Software and code

Policy information about availability of computer code

Data collection	Data collection for flow cytometry stainings was performed employing BD FACSDiva (Version 8.0.2) and FlowJo software (Version 10.4.2)
Data analysis	Data analysis was performed in a local installation of Galaxy (version 21.09) maintained by the EMBL Genome Biology Computational Support and in R (versions 3.6 and 4.2) using Bioconductor packages. For all sequencing assays, the quality of the sequencing run was assessed using FastQC (v 0.11.5) and adapter sequences were trimmed using TrimGalore (v 0.4.3).<br>For low-coverage DNA-seq analysis, sequencing reads were aligned to the mouse reference genome (GRCm38/mm10 assembly) using Bowtie2 (v2.3.4) with default settings and uniquely mapped reads (MAPQ ≥ 30) were retained for the subsequent steps. The bam files thus generated were indexed and used for copy number variation analysis (bin size of 100 kb) using the Coral script (https://github.com/tobiasrausch/coral).<br>For mRNA-seq analysis, sequencing reads were aligned to the mouse reference genome (GRCm38/mm10 assembly) using STAR (v2.5.2b) with default settings and uniquely mapped reads (MAPQ ≥ 20) were retained for the subsequent steps. Gene count tables were generated with featureCounts (subread v1.6.2), using gencode gene annotations (release M10). Coverage files were generated using bamCoverage (deeptools v2.4.1), and the differential expression analysis was performed in R using the DESeq2 package. Genes were considered differentially expressed using a false discovery rate (FDR) cut-off of 0.05. Gene ontology enrichment analysis was conducted using ClusterProfiler and plots were generated using ggplot2 and ggpubr R packages.<br>For ChIP-seq analysis, sequencing reads were aligned to the mouse reference genome (GRCm38/mm10 assembly) using Bowtie2 (v2.3.4). Uniquely mapped reads (MAPQ ≥ 20) aligning to major chromosomes were retained for the subsequent steps. ChIP signal strength and sequencing depth were assessed using the plotFingerprint and plotCoverage (deeptools v2.4.1). Coverage files (bigwig format) were generated using deeptools bamCoverage: 10 bp was used as bin size, the “reads per genomic content (RPGC)” method was used for normalization and reads were extended to an average fragment size of 150 bp. Peak calling was performed with MACS2, by providing ChIP and respective input bam files and considering a minimum FDR cut-off for peak detection of 0.05. Differential peak analysis was performed in

R using the DiffBind package, only peaks detected in at least two replicates were retained for the analysis and peaks were called as significantly differential considering an FDR cut-off = 0.05. Heatmaps and metagene plots were generated in R with custom scripts and using the ggplot2 and pheatmap packages.
For PRO-seq analysis, pre-processing and alignment were performed using the PROseq2.0 pipeline (https://github.com/Danko-Lab/proseq2.0). The consensus set of distal cis-regulatory elements active at the basal state in mESCs was obtained by running the dREG package (https://github.com/Danko-Lab/dREG) on the PRO-seq data from control cell lines. The differential PRO-seq analysis was performed using the output of the dREG package from control and H3.3K9A lines as input for the tfTarget package (https://github.com/Danko-Lab/tfTarget). The violin plots were generated by calculating RPKM values for mutant and control samples within the genomic regions of interest.

For histone modifications mass-spectrometry analysis, data were analyzed using a combination of EpiProfile 2.0, Skyline, and manual analysis with Xcalibur.

Analysis of flow cytometry data was performed using FlowJo software (v10.4.2).

For manuscripts utilizing custom algorithms or software that are central to the research but not yet described in published literature, software must be made available to editors and reviewers. We strongly encourage code deposition in a community repository (e.g. GitHub). See the Nature Portfolio guidelines for submitting code & software for further information.

Data

Policy information about availability of data

All manuscripts must include a data availability statement. This statement should provide the following information, where applicable:

- Accession codes, unique identifiers, or web links for publicly available datasets
- A description of any restrictions on data availability
- For clinical datasets or third party data, please ensure that the statement adheres to our policy

Next-Generation Sequencing datasets (DNA-seq, mRNA-seq, ChIP-seq, and PRO-seq) have been deposited in the ArrayExpress database with the following accession numbers: E-MTAB-12866 (mRNA-seq), E-MTAB-12867 (DNA-seq), E-MTAB-12868 (ChIP-seq), and E-MTAB-12869 (PRO-seq). Following links can be used to access the datasets before they are made public:
- E-MTAB-12866: https://tinyurl.com/2ksnwwdx
- E-MTAB-12867: https://tinyurl.com/2p8pwt3f
- E-MTAB-12868: https://tinyurl.com/2p8sv958
- E-MTAB-12869: https://tinyurl.com/4dndk9xz

Research involving human participants, their data, or biological material

Policy information about studies with human participants or human data. See also policy information about sex, gender (identity/presentation), and sexual orientation and race, ethnicity and racism.

Reporting on sex and gender

Use the terms sex (biological attribute) and gender (shaped by social and cultural circumstances) carefully in order to avoid confusing both terms. Indicate if findings apply to only one sex or gender; describe whether sex and gender were considered in study design; whether sex and/or gender was determined based on self-reporting or assigned and methods used.
Provide in the source data disaggregated sex and gender data, where this information has been collected, and if consent has been obtained for sharing of individual-level data; provide overall numbers in this Reporting Summary. Please state if this information has not been collected.
Report sex- and gender-based analyses where performed, justify reasons for lack of sex- and gender-based analysis.

Reporting on race, ethnicity, or other socially relevant groupings

Please specify the socially constructed or socially relevant categorization variable(s) used in your manuscript and explain why they were used. Please note that such variables should not be used as proxies for other socially constructed/relevant variables (for example, race or ethnicity should not be used as a proxy for socioeconomic status).
Provide clear definitions of the relevant terms used, how they were provided (by the participants/respondents, the researchers, or third parties), and the method(s) used to classify people into the different categories (e.g. self-report, census or administrative data, social media data, etc.)
Please provide details about how you controlled for confounding variables in your analyses.

Population characteristics

Describe the covariate-relevant population characteristics of the human research participants (e.g. age, genotypic information, past and current diagnosis and treatment categories). If you filled out the behavioural & social sciences study design questions and have nothing to add here, write "See above."

Recruitment

Describe how participants were recruited. Outline any potential self-selection bias or other biases that may be present and how these are likely to impact results.

Ethics oversight

Identify the organization(s) that approved the study protocol.

Note that full information on the approval of the study protocol must also be provided in the manuscript.

Field-specific reporting

Please select the one below that is the best fit for your research. If you are not sure, read the appropriate sections before making your selection.

☒ Life sciences ☐ Behavioural & social sciences ☐ Ecological, evolutionary & environmental sciences

Life sciences study design

All studies must disclose on these points even when the disclosure is negative.

Sample size	Sample size for each experiment is indicated in the figure legend for each experiment. Details about the number of analyzed genomic features (n) used to generate plots from sequencing data are depicted in the respective figure, or corresponding figure caption.<br>mRNA-seq samples were sequenced to an approximate sequencing depth of 25-30 million uniquely mapped reads to reach sufficient coverage as recommended by the ENCODE consortium (2011, https://www.encodeproject.org/) and Liu et al., 2014 (PMID: 24319002).<br>ChIP-seq samples were sequenced to an approximate sequencing depth of 15-30 million uniquely mapped reads to reach sufficient coverage as recommended by the ENCODE consortium (2011, https://www.encodeproject.org/) and Landt et al., 2012 (PMID: 22955991).<br>PRO-seq samples were sequenced to an approximate depth of 30-45 million uniquely mapped reads to reach sufficient coverage as recommended by Mahat et al., 2016 (PMID: 27442863).<br>Sample sizes of mRNA-seq, ChIP-seq and PRO-seq experiments were chosen in agreement with guidelines for the analysis of next-generation sequencing data and to fulfill the requirements of published bioinformatic tools employed in this study (n=3).
Data exclusions	Cell lines were screened for genomic integrity after CRISPR-Cas9 targeting (i.e., chromosome loss or chromosome duplication) and only mESC clones that showed no impaired were used in this study.<br>The sequencing data were subjected to preliminary quality assessments as recommended by the ENCODE consortium and established by the EMBL Genome Biology Computational Support. No data was excluded from mRNA-seq, ChIP-seq and PRO-seq experiments.
Replication	All experimental findings were reliably reproducible. Reproducibility between biological replicates used for sequencing experiments was assessed by hierarchical clustering and PCA analysis.
Randomization	No randomization was required for this cell-culture based in vitro study. All test cell lines were generated from a common parental mouse embryonic stem cell line.
Blinding	In this in vitro study no group allocation was done.

Reporting for specific materials, systems and methods

We require information from authors about some types of materials, experimental systems and methods used in many studies. Here, indicate whether each material, system or method listed is relevant to your study. If you are not sure if a list item applies to your research, read the appropriate section before selecting a response.

Materials & experimental systems

n/a	Involved in the study	
☐	☒	Antibodies
☐	☒	Eukaryotic cell lines
☒	☐	Palaeontology and archaeology
☒	☐	Animals and other organisms
☒	☐	Clinical data
☒	☐	Dual use research of concern
☒	☐	Plants

Methods

n/a	Involved in the study	
☐	☒	ChIP-seq
☐	☒	Flow cytometry
☒	☐	MRI-based neuroimaging

Antibodies

Antibodies used	H3.3 (09-838 – Merck-Millipore)<br>H3K27ac (39685 – Active Motif)<br>H3K27me3 (C36B11 – Cell Signalling Technology)<br>H3K9ac (C5B11-9549 – Cell Signalling Technology)<br>H3K9me3 (D4W1U – Cell Signalling Technology)<br>H3K9me2 (D85B4 – Cell Signalling Technology)<br>H3K18ac (9675 – Cell Signalling Technology)<br>SUZ12 (D39F6-3737 – Cell Signalling Technology)<br><br>PE-CD59a (143103 – Biolegend)<br>APC-TLR2/CD282 (153005 – Biolegend)<br>FITC-MHC Class II (107605 – Biolegend)<br>PECy7-CD11b (561098 – BD)<br>BV605-F4/80 (123133 – Biolegend)
Validation	All used antibodies are commercially available and have been validated by the manufacturer. For ChIP-seq, the antibodies used were specified as suitable for this application by the manufacturer.<br>Antibody validations and validation criteria are available on these websites:

https://www.activemotif.com/catalog/802/chip-seq-validated-antibodies
https://www.cellsignal.com/about-us/cst-antibody-validation-principles

Eukaryotic cell lines

Policy information about cell lines and Sex and Gender in Research

Cell line source(s)	All experiments were performed using murine embryonic stem cells (ESCs) (129XC57BL/6J), generated from male 129-B13 agouti mice.
Authentication	Genome editing and genome integrity in mouse ESCs was confirmed by low-coverage DNA-seq analysis and comparison to the reference genome (mm10).
Mycoplasma contamination	All cell lines were routinely tested negative for mycoplasma contamination.
Commonly misidentified lines (See ICLAC register)	N/A

Plants

Seed stocks	*Report on the source of all seed stocks or other plant material used. If applicable, state the seed stock centre and catalogue number. If plant specimens were collected from the field, describe the collection location, date and sampling procedures.*
Novel plant genotypes	*Describe the methods by which all novel plant genotypes were produced. This includes those generated by transgenic approaches, gene editing, chemical/radiation-based mutagenesis and hybridization. For transgenic lines, describe the transformation method, the number of independent lines analyzed and the generation upon which experiments were performed. For gene-edited lines, describe the editor used, the endogenous sequence targeted for editing, the targeting guide RNA sequence (if applicable) and how the editor was applied.*
Authentication	*Describe any authentication procedures for each seed stock used or novel genotype generated. Describe any experiments used to assess the effect of a mutation and, where applicable, how potential secondary effects (e.g. second site T-DNA insertions, mosiacism, off-target gene editing) were examined.*

ChIP-seq

Data deposition

☒ Confirm that both raw and final processed data have been deposited in a public database such as GEO.

☐ Confirm that you have deposited or provided access to graph files (e.g. BED files) for the called peaks.

Data access links<br>*May remain private before publication.*	All raw ChIP-seq data are deposited in Array Express under accession number E-MTAB-12868. The following link can be used to access the data: https://tinyurl.com/2p8sv958
Files in database submission	H3.3_Ctrl_KoA27N<br>H3.3_Ctrl_KoA313<br>H3.3_Ctrl_KoA317<br>H3.3_K27A_E6<br>H3.3_K27A_G9<br>H3.3_K27A_G9_2b<br>H3.3_K9A_A4<br>H3.3_K9A_H5<br>H3.3_K9A_H5_9b<br>H3K27ac_Ctrl_27N2<br>H3K27ac_Ctrl_313<br>H3K27ac_Ctrl_317<br>H3K27ac_K27A_C2<br>H3K27ac_K27A_E6<br>H3K27ac_K27A_G9<br>H3K27ac_K79A_c52<br>H3K27ac_K79A_cl5<br>H3K27ac_K79A_E126<br>H3K27ac_K9A_A2<br>H3K27ac_K9A_A4<br>H3K27ac_K9A_H5<br>H3K27me3_Ctrl_27N<br>H3K27me3_Ctrl_313<br>H3K27me3_Ctrl_313_9b<br>H3K27me3_K27A_C2<br>H3K27me3_K27A_E6<br>H3K27me3_K27A_G9<br>H3K27me3_K9A_A2<br>H3K27me3_K9A_A4

H3K27me3_K9A_H5
H3K9ac_Ctrl_27N2
H3K9ac_Ctrl_313
H3K9ac_Ctrl_317
H3K9ac_K27A_C2
H3K9ac_K27A_E6
H3K9ac_K27A_G9
H3K9ac_K9A_A2
H3K9ac_K9A_A4
H3K9ac_K9A_H5
H3K9me2_Ctrl_27N
H3K9me2_Ctrl_313
H3K9me2_Ctrl_313J
H3K9me2_K27A_C2
H3K9me2_K27A_E6
H3K9me2_K27A_G9
H3K9me2_K9A_A2
H3K9me2_K9A_A4
H3K9me2_K9A_H5
H3K9me3_Ctrl_KoA27N
H3K9me3_Ctrl_KoA313
H3K9me3_Ctrl_KoA317
H3K9me3_K27A_E6
H3K9me3_K27A_G9
H3K9me3_K9A_A4
H3K9me3_K9A_H5
Input_Ctrl_313
Input_Ctrl_koa313
Input_Ctrl_KoA313
Input_K27A_C2
Input_K27A_E6
Input_K27A_G9
Input_K79A_E126
Input_K9A_A2
Input_K9A_A4
Input_K9A_H5
Suz12_Ctrl_27N
Suz12_Ctrl_313
Suz12_Ctrl_313_9b
Suz12_K27A_C2
Suz12_K27A_E6
Suz12_K27A_G9
Suz12_K9A_A2
Suz12_K9A_A4
Suz12_K9A_H5

Genome browser session
(e.g. UCSC)

https://tinyurl.com/Trovatoetal2024UCSC

Methodology

Replicates

Three biological replicates were used for H3.3, H3K27ac, H3K27me3, H3K9ac, H3K9me2 and SUZ12 ChIP-seq experiments. Three biological replicates for control mESCs and two biological replicates for mutant mESCs were used for H3K9me3 ChIP-seq experiment.

Sequencing depth

Libraries were sequenced either on Illumina's NextSeq500 (75 bp single-end mode) or NextSeq2000 (P3 kit – 88 bp in single-end mode). The number of total/non-duplicated uniquely aligned reads for each sample is reported below:
H3.3_Ctrl_KoA27N: 28145974/21939173
H3.3_Ctrl_KoA313: 28248955/21083686
H3.3_Ctrl_KoA317: 30428883/22879544
H3.3_K27A_E6: 24980943/9185604
H3.3_K27A_G9: 29064480/22113974
H3.3_K27A_G9_2b: 26162473/19910575
H3.3_K9A_A4: 28717681/20584884
H3.3_K9A_H5: 27989879/19972999
H3.3_K9A_H5_9b: 24599898/17932846
H3K27ac_Ctrl_27N2: 31472710/22208175
H3K27ac_Ctrl_313: 33231101/21838828
H3K27ac_Ctrl_317: 39682821/26210570
H3K27ac_K27A_C2: 23709224/17040105
H3K27ac_K27A_E6: 33916813/25077326
H3K27ac_K27A_G9: 29112288/20753200
H3K27ac_K79A_c52: 37439256/26330571
H3K27ac_K79A_cl5: 32923518/23345965
H3K27ac_K79A_E126: 29963793/21251128
H3K27ac_K9A_A2: 35609520/23347286
H3K27ac_K9A_A4: 32802543/20897175

H3K27ac_K9A_H5: 32011644/20166905
H3K27me3_Ctrl_27N: 35941559/22043081
H3K27me3_Ctrl_313: 36742103/22650961
H3K27me3_Ctrl_313_9b: 41337123/24565891
H3K27me3_K27A_C2: 34227079/18828732
H3K27me3_K27A_E6: 37092441/19696970
H3K27me3_K27A_G9: 36554788/19651570
H3K27me3_K9A_A2: 44213206/27313620
H3K27me3_K9A_A4: 45838138/28141926
H3K27me3_K9A_H5: 39288374/24690723
H3K9ac_Ctrl_27N2: 30309841/25423443
H3K9ac_Ctrl_313: 33206124/27925402
H3K9ac_Ctrl_317: 26466974/22409415
H3K9ac_K27A_C2: 31632794/26990119
H3K9ac_K27A_E6: 28078044/23970205
H3K9ac_K27A_G9: 33484471/28469592
H3K9ac_K9A_A2: 33325149/26636098
H3K9ac_K9A_A4: 30229750/24286330
H3K9ac_K9A_H5: 34068674/27879672
H3K9me2_Ctrl_27N: 45389405/29635966
H3K9me2_Ctrl_313: 40926082/25597674
H3K9me2_Ctrl_313J: 50612346/30810638
H3K9me2_K27A_C2: 42610760/27444822
H3K9me2_K27A_E6: 44756317/28545881
H3K9me2_K27A_G9: 45947371/29725645
H3K9me2_K9A_A2: 47018664/30286929
H3K9me2_K9A_A4: 46857488/29808894
H3K9me2_K9A_H5: 43286079/28530807
H3K9me3_Ctrl_KoA27N: 29463513/11996423
H3K9me3_Ctrl_KoA313: 32360691/14060132
H3K9me3_Ctrl_KoA317: 33136834/14277247
H3K9me3_K27A_E6: 25155976/9185604
H3K9me3_K27A_G9: 32359482/12544972
H3K9me3_K9A_A4: 28433804/12774475
H3K9me3_K9A_H5: 31402528/13926984
Input_Ctrl_313: 50185972/29891045
Input_Ctrl_koa313: 50571337/38285402
Input_Ctrl_KoA313: 32643792/24149323
Input_K27A_C2: 36750642/22137186
Input_K27A_E6: 41424027/31629131
Input_K27A_G9: 29666592/21877610
Input_K79A_E126: 47848791/36629967
Input_K9A_A2: 38826752/29394313
Input_K9A_A4: 28727676/11622968
Input_K9A_H5: 26439453/19377254
Suz12_Ctrl_27N: 47173037/27763757
Suz12_Ctrl_313: 41693596/24702700
Suz12_Ctrl_313_9b: 47863839/28430843
Suz12_K27A_C2: 51153729/29548272
Suz12_K27A_E6: 46346919/27320351
Suz12_K27A_G9: 41866389/22729457
Suz12_K9A_A2: 48571841/28593301
Suz12_K9A_A4: 48104722/27884685
Suz12_K9A_H5: 44934466/25427722

Antibodies

H3.3 (09-838 – Merck-Millipore)
H3K27ac (39685 – Active Motif)
H3K27me3 (C36B11 – Cell Signalling Technology)
H3K9ac (C5B11-9549 – Cell Signalling Technology)
H3K9me3 (D4W1U – Cell Signalling Technology)
H3K9me2 (D85B4 – Cell Signalling Technology)
SUZ12 (D39F6-3737 – Cell Signalling Technology)

Peak calling parameters

Sequencing reads were aligned to mouse reference genome (mm10 assembly) using Bowtie2. Uniquely mapped reads (MAPQ ≥ 20) aligning to major chromosomes were retained for the subsequent steps.
Peaks were called with MACS2 (Zhang et al.), by providing ChIP and respective input bam files and considering a minimum FDR cut-off for peak detection of 0.05; narrow or broad peak calling was performed for histone modifications following ENCODE guidelines (https://www.encodeproject.org/chip-seq/histone/).

Data quality

Data quality for both the uniquely aligned reads and all sequencing reads was assessed using FastQC tool. Unmapped reads or reads mapping at more than one location were removed. Read duplicates were identified and removed using Picard's Mark Duplicates. Reproducibility between ChIP-seq replicates was assessed by PCA analysis and samples correlation on binned and library size normalized bigwig files using deeptools. Number of peaks (FDR=0.05) for each sample are reported below:
H3.3_Ctrl_KoA27N: 123933
H3.3_Ctrl_KoA313: 118702

H3.3_Ctrl_KoA317: 139055
H3.3_K27A_E6: 108106
H3.3_K27A_G9: 86677
H3.3_K27A_G9_2b: 119644
H3.3_K9A_A4: 64967
H3.3_K9A_H5: 72889
H3.3_K9A_H5_9b: 141487
H3K27ac_Ctrl_27N2: 90446
H3K27ac_Ctrl_313: 87987
H3K27ac_Ctrl_317: 84419
H3K27ac_K27A_C2: 66698
H3K27ac_K27A_E6: 68277
H3K27ac_K27A_G9: 63017
H3K27ac_K79A_c52: 86023
H3K27ac_K79A_cl5: 82389
H3K27ac_K79A_E126: 93775
H3K27ac_K9A_A2: 77915
H3K27ac_K9A_A4: 87635
H3K27ac_K9A_H5: 83019
H3K27me3_Ctrl_27N: 39949
H3K27me3_Ctrl_313: 42122
H3K27me3_Ctrl_313_9b: 47194
H3K27me3_K27A_C2: 31166
H3K27me3_K27A_E6: 30889
H3K27me3_K27A_G9: 32176
H3K27me3_K9A_A2: 18627
H3K27me3_K9A_A4: 24794
H3K27me3_K9A_H5: 28565
H3K9ac_Ctrl_27N2: 86538
H3K9ac_Ctrl_313: 86716
H3K9ac_Ctrl_317: 64547
H3K9ac_K27A_C2: 62857
H3K9ac_K27A_E6: 59162
H3K9ac_K27A_G9: 66099
H3K9ac_K9A_A2: 103935
H3K9ac_K9A_A4: 90791
H3K9ac_K9A_H5: 103895
H3K9me2_Ctrl_27N: N/A
H3K9me2_Ctrl_313: N/A
H3K9me2_Ctrl_313J: N/A
H3K9me2_K27A_C2: N/A
H3K9me2_K27A_E6: N/A
H3K9me2_K27A_G9: N/A
H3K9me2_K9A_A2: N/A
H3K9me2_K9A_A4: N/A
H3K9me2_K9A_H5: N/A
H3K9me3_Ctrl_KoA27N: 52988
H3K9me3_Ctrl_KoA313: 66950
H3K9me3_Ctrl_KoA317: 67412
H3K9me3_K27A_E6: 34240
H3K9me3_K27A_G9: 56779
H3K9me3_K9A_A4: 25445
H3K9me3_K9A_H5: 27301
Suz12_Ctrl_27N: 3026
Suz12_Ctrl_313: 4085
Suz12_Ctrl_313_9b: 4460
Suz12_K27A_C2: 2441
Suz12_K27A_E6: 2388
Suz12_K27A_G9: 2817
Suz12_K9A_A2: 2801
Suz12_K9A_A4: 2835
Suz12_K9A_H5: 3366

Software

Quality assessment and pre-processing were performed in a local installation of Galaxy maintained by the EMBL Genome Biology Computational Support. Sequencing reads were aligned to the mouse reference genome (GRCm38/mm10 assembly) using Bowtie2 (v2.3.4). Uniquely mapped reads (MAPQ ≥ 20) aligning to major chromosomes were retained for the subsequent steps. ChIP signal strength and sequencing depth were assessed using the plotFingerprint and plotCoverage (deeptools v2.4.1). Coverage files (bigwig format) were generated using deeptools bamCoverage: 10 bp was used as bin size, the “reads per genomic content (RPGC)” method was used for normalization and reads were extended to an average fragment size of 150 bp. Differential peak analysis was performed in R using the DiffBind package, only peaks detected in at least two replicates were retained for the analysis and peaks were called as significantly differential considering an FDR cut-off = 0.05. Annotation of ChIP-seq peaks was performed with the ChIPseeker package and with publicly available ChromHMM state maps for mESCs (https://github.com/guifengwei/ChromHMM_mESC_mm10). Genomic coordinates of promoters of protein coding genes were retrieved from ENSEMBL using biomart (mm10 version: https://nov2020.archive.ensembl.org). Heatmaps and metagene plots were generated in R; Boxplots and violin plots were generated considering the ChIP-seq signal measured at TSS or peak summit ± 1 kb.

Flow Cytometry

Plots

Confirm that:

☒ The axis labels state the marker and fluorochrome used (e.g. CD4-FITC).

☒ The axis scales are clearly visible. Include numbers along axes only for bottom left plot of group (a 'group' is an analysis of identical markers).

☒ All plots are contour plots with outliers or pseudocolor plots.

☒ A numerical value for number of cells or percentage (with statistics) is provided.

Methodology

Sample preparation	Cell cycle analysis: the percentage of proliferating cells in S-Phase was measured using the EdU Click FC-488 Kit (Carl Roth – BA-7779) following the manufacturer's instructions. Briefly, EdU was added at a final concentration of 10 µM and cells were incubated for 2.5 hours at 37°C. Cells were washed once with PBS and then dissociated using accutase. Cells were fixed by resuspension in 100 µl (every 10^6) of 4% PFA in PBS and incubation 15 minutes at RT, in the dark. After quenching with PBS + 1% BSA, cells were centrifuged and the pellet resuspended in 1X saponin-based permeabilization buffer. A total of 4.5x10^5 cells were used for each condition and 500 µl of Click reaction master mix (PBS, catalyst solution, dye azide and 10X buffer additive) were added to each tube. After 30 minutes incubation at RT in the dark, cells were washed once with 3 ml of saponin-based permeabilization buffer and DNA content was stained with propidium iodide (7 minutes incubation at RT). Cells were washed once with saponin-based permeabilization buffer and resuspended in 300-500 µl of buffer and analyzed by flow cytometry. Three controls were included for flow cytometry analysis: i) w/o EdU, w/o click and w/o DAPI; ii) w/o Edu, with click and DAPI; iii) with EdU, w/o click and with DAPI.<br><br>Cell death assay: The FITC Annexin V Kit (BD Pharmingen – 556419) was used, which allows to detect earlier stages of cell death, preceding loss of membrane integrity. Briefly, cells were washed twice with PBS and resuspended in 1X binding buffer to a concentration of 10^6 cells/ml. To 100 µl of the mix (~ 10^5 cells), 5 µl of FITC Annexin V and 10 µl of propidium iodide (50 µg/ml stock) were added and cells were incubated for 15 minutes at RT, in the dark. After incubation, 400 µl of 1X binding buffer were added to each tube and cells were analyzed by flow cytometry. Three controls were included for flow cytometry analysis: i) unstained cells; ii) cells with FITC Annexin V, w/o PI; iii) cells w/o FITC-Annexin V, with PI.<br><br>Immune proteins staining: cells were harvested and washed with FACS buffer (PBS + 2% FBS). Cells were then resuspended in 100 µl of master mix composed of FACS buffer and desired antibodies, each one diluted 1:100. Alternatively, cells were resuspended in FACS buffer only for the unstained controls. After 30 minutes incubation on ice and in the dark, cells were centrifuged and washed with FACS buffer. Antibodies used for the staining were the following: PE-CD59a (143103 – Biolegend); APC-TLR2/CD282 (153005 – Biolegend); FITC-MHC Class II (107605 – Biolegend); PECy7-CD11b (561098 – BD); BV605-F4/80 (123133 – Biolegend).<br><br>Bodipy-C11 staining: the staining was performed following the guidelines described by Martinez et al., 2020. In brief, the BODIPY-C11 probe was added at a final concentration of 2.5 µM and cells were incubated for 30 minutes at 37°C. Cells were then washed with HBSS buffer, collected and analyzed by flow cytometry.
Instrument	BD LSR Fortessa<br>BD FACSAria
Software	FlowJo
Cell population abundance	Percentages of cell populations for each condition were quantified and statistically analyzed and are depicted as barplots/ boxplots.
Gating strategy	Initial gating was set for all samples based on physical parameters (forward scatter-area (FSC-A) vs. side scatter-area (SSC-A); P1) to exclude dead cells and debris from live cells. Next, singlets were selected based on SSC-A vs. SSC-W (P2; Bodipy C11 and apoptosis assays) or assessing the DNA content through the DAPI channel (405-450/50-A vs. 405-450/50-H; EdU cell-cycle assay). For the cell-cycle assay, EdU incorporation was acquired on the FITC channel (488-530/30-A; logarithmic) and the DNA content on the DAPI channel (405-450/50-A; linear) channel. For the Bodipy C11 assay, the same initial physical gating strategies (P1 and P2) were applied. Lipid peroxidation was acquired on the SSC and FITC channels (SSC-A vs. 488-530/30-A). For the apoptosis assay, we followed the same initial physical gating strategy (P1 and P2). Discrimination of live, early apoptotic, necrotic and late apoptotic cells was performed based on the intensities of Alexa Fluor-488 (FITC channel: 488-530/30-A) and propidium iodide stainings (561-610/20-A) as follows: Live cells: Alexa Fluor-488-dim/propidium iodide-dim; early apoptotic cells: Alexa Fluor-488-dim/propidium iodide-high; necrotic cells: Alexa Fluor-488-high/propidium iodide-dim; late apoptotic cells: Alexa Fluor-488-high/propidium iodide-high.<br>For immune proteins staining, target cells were identified based on their forward and side scatter areas (FSC-A versus SSC-A), and debris were gated out. Doublets were excluded by plotting FSC-A versus FSC-hight (FSC-H), and SSC-width (SSC-W) versus SSC-hight (SSC-H). Viable cells only were considered for the flow cytometry-based analyses, and identified as the cell population negative for the dye DAPI (FSC-A versus DAPI-A). Unstained cells were used as negative control to gate the cell population positive for each marker staining. The median fluorescence intensity (MFI) values were used for the analyses. For each sample 10.000 events were recorded.

☒ Tick this box to confirm that a figure exemplifying the gating strategy is provided in the Supplementary Information.
